# Method of Calculating the Inductance Value of MEMS Suspended Inductors with Silicon Substrates

**DOI:** 10.3390/mi9110604

**Published:** 2018-11-17

**Authors:** Yiyuan Li, Jianhua Li, Lixin Xu

**Affiliations:** School of Mechatronical Engineering, Beijing Institute of Technology, Beijing 100081, China; lyy510@bit.edu.cn (Y.L.); lxxu@bit.edu.cn (L.X.)

**Keywords:** microelectromechanical system (MEMS), MEMS suspended inductor, inductance value

## Abstract

Microelectromechanical system (MEMS) suspended inductors have excellent radio-frequency (RF) performance. The inductance value is one of the main features that characterizes the performance of inductors. It is important to consider the influence of the substrate and the suspension height in calculating the inductance value accurately. In this paper, a method is proposed to calculate the inductance value of the MEMS suspended inductor wire with a silicon substrate, as the wire is the basic component of the inductor coil. Then the method is extended to the suspended inductors consisting of a single turn coil. The calculation results obtained by this proposed method were verified by finite-element analysis (HFSS) and they were found to agree well with the results of the HFSS simulation.

## 1. Introduction

Microelectromechanical system (MEMS) planar spiral inductors have been widely studied in recent decades due to their benefits in improving the performance of radio frequency integrated circuits (RFICs) [1,2,3]. MEMS suspended inductors can even achieve a better radio-frequency performance because the substrate loss is reduced by lifting the inductor coil several micrometers above the silicon substrate [4,5,6]. However, MEMS suspended inductors have poor mechanical properties. The suspended structures are susceptible to deforming under mechanical shock during their fabrication, shipping and operation. As the shock-sensitive direction of the suspended inductor is perpendicular to the plane of the coil, the deformation of the suspended inductor is reflected in the change of the suspension height, which is the distance between the coil and the substrate. The inductance value is one of the main features that characterizes the performance of inductors [4]. The inductance value of the MEMS suspended inductor will change as the suspension height changes when the suspended inductor is shocked.

Many studies have been carried out to calculate the inductance value of the planar spiral inductors. Greenhouse presented a method to calculate the inductance value of planar rectangular microelectronics inductors based on the physical concept of the inductors [7]. Mohan introduced a fitting formula to calculate the planar inductors with its geometry parameters [8]. Although the inductance value of the inductor coil can be calculated, these methods did not take into account the influence of the substrate on the inductance value. As for the MEMS suspended inductor, the silicon substrate, the suspension height and the frequency also have an influence on its inductance value. Yue et al. presented a π model for a planar on-chip inductor [9], but for the MEMS suspended inductor this model lacks consideration of the influence of the air layer between the coil and the substrate. Furthermore, the model parameters describing the substrate parasitics need to be obtained by simulation or measurement [9,10,11,12]. When the suspended inductor is under shock, the suspension height is changed, thus it is hard to calculate the inductance value with the π model. Therefore few methods can be used to calculate the inductance value of the MEMS suspended inductor with silicon substrate.

The magnetic flux, which is required to calculate the inductance value, can be obtained by solving the electromagnetic field generated by the inductor wires and coils in the air layer. Thus calculating the inductance value of the MEMS suspended inductor with a silicon substrate can be considered as a problem of solving electromagnetic fields in stratified media. King et al. studied such problems in the 1990s. They presented approximate formulas for the electromagnetic fields of vertical electric dipoles and horizontal electric dipoles, in the presence of a three-layered region [13,14,15]. Then several studies improved King’s theory by considering the trapped surface wave that was previously ignored [16,17,18,19].

The wire is the basic component of the MEMS suspended inductor coil. This paper presents a method that can be used to calculate the inductance value of the MEMS suspended inductor wire with a silicon substrate. The influence of the silicon substrate and the suspension height are considered. When the deformation variation of the MEMS suspended inductors during shock is obtained by calculation or simulation, the variation of the inductance value can be calculated directly using the method in this paper. The inductor wire was considered a series of electric dipoles. By superimposing the electromagnetic fields generated by these electric dipoles, the magnetic flux and the inductance value of the inductor were obtained. Then the method was extended to the MEMS suspended inductor consisting of a single turn coil with a silicon substrate. Finally, the ANSYS HFSS software (Canonsburg, PA, USA) was used to verify the results calculated using this method.

## 2. Inductance of the Suspended Inductors on a Silicon Substrate

The MEMS suspended inductor consisted of copper wires in an air layer, silicon substrate (dielectric layer) and ground (perfect conductor layer). For the MEMS suspended inductor, the width w and thickness t of the wires were only in the order of 10 μm, and the length of the wires was in the order of 100 μm. When the frequency was 10 GHz, the wavelength was 3 cm. Thus the length, width and thickness of the inductor wires were much smaller than the wavelength. The inductor wires were considered as a series of electric dipoles.

### 2.1. Calculation of the Inductance Value of the Suspended Inductor Consisting of a Single Wire

**Figure 1:**
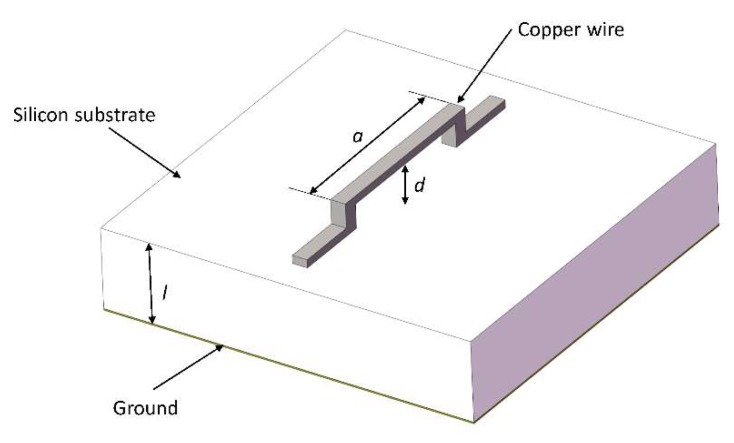
The schematic of the microelectromechanical system (MEMS) suspended inductor consisting of a single wire.

**Figure 2:**
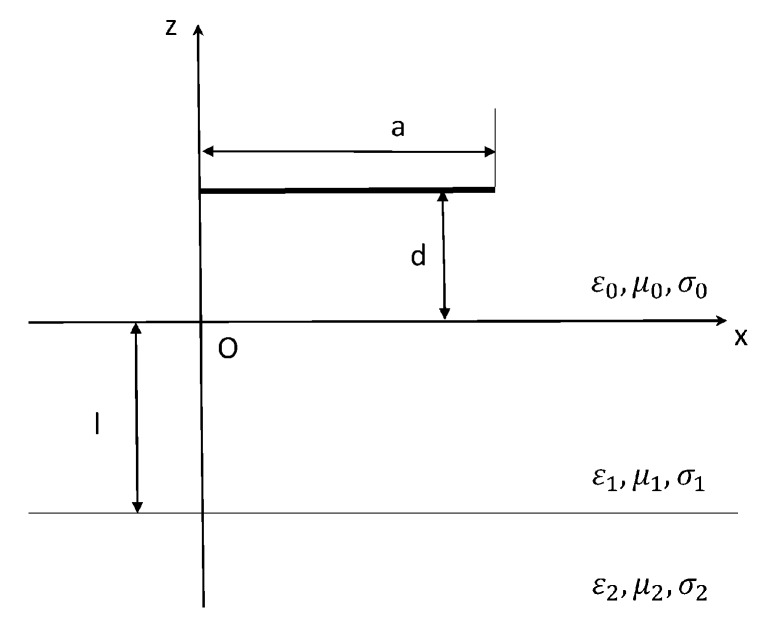
Geometry of a wire at a height of d over the substrate.

The schematic of the MEMS suspended inductor consisting of a single wire is shown in Figure 1 and the geometry under consideration is shown in Figure 2.

As Figure 2 shows, a copper wire is located at the height d above the silicon substrate. The length of the wire is a and the thickness of the substrate is l. In Figure 2, region 0 (z>0) indicates the air layer above the substrate, region 1 (−l≤z≤0) indicates the silicon substrate, and region 2 (z<−l) indicates the ground. The permittivity, permeability and conductivity of the region i are expressed as $\varepsilon_{i},\;\mu_{i},\;\sigma_{i}$ in Figure 2.

In the cylindrical coordinates, the integral expressions of the electromagnetic field components in region 0, due to a horizontal electric dipole at (0, 0, d), can be expressed as:

(1) $$\;E_{0\rho }\left(\rho ,\varphi ,z\right)=-\frac{\omega \mu_{0}Idl}{4\pi k_{0}{}^{2}}\cos\varphi \left[F_{\rho 0}\left(\rho ,z-d\right)-F_{\rho 0}\left(\rho ,z+d\right)+F_{\rho 1}\left(\rho ,z+d\right)+F_{\rho 2}\left(\rho ,z+d\right)\right]$$

(2) $$\;E_{0\varphi }\left(\rho ,\varphi ,z\right)=\frac{\omega \mu_{0}Idl}{4\pi k_{0}{}^{2}}\sin\varphi \left[F_{\varphi 0}\left(\rho ,z-d\right)-F_{\varphi 0}\left(\rho ,z+d\right)+F_{\varphi 1}\left(\rho ,z+d\right)+F_{\varphi 2}\left(\rho ,z+d\right)\right]$$

(3) $$\;E_{0z}\left(\rho ,\varphi ,z\right)=\frac{i\omega \mu_{0}Idl}{4\pi k_{0}{}^{2}}\cos\varphi \left[F_{z0}\left(\rho ,z-d\right)-F_{z0}\left(\rho ,z+d\right)+F_{z1}\left(\rho ,z+d\right)\right]$$

(4) $$B_{0\rho }\left(\rho ,\varphi ,z\right)=-\frac{\mu_{0}Idl}{4\pi }\sin\varphi \left[G_{\rho 0}\left(\rho ,z-d\right)-G_{\rho 0}\left(\rho ,z+d\right)+G_{\rho 1}\left(\rho ,z+d\right)+G_{\rho 2}\left(\rho ,z+d\right)\right]$$

(5) $$B_{0\varphi }\left(\rho ,\varphi ,z\right)=-\frac{\mu_{0}Idl}{4\pi }\cos\varphi \left[G_{\varphi 0}\left(\rho ,z-d\right)-G_{\varphi 0}\left(\rho ,z+d\right)+G_{\varphi 1}\left(\rho ,z+d\right)+G_{\varphi 2}\left(\rho ,z+d\right)\right]$$

(6) $$\;B_{0z}\left(\rho ,\varphi ,z\right)=\frac{i\mu_{0}Idl}{4\pi }\sin\varphi \left[G_{z0}\left(\rho ,z-d\right)-G_{z0}\left(\rho ,z+d\right)+G_{z2}\left(\rho ,z+d\right)\right]\;$$

where $k_{i}$ is the wave number in region *i* and $k_{i}$ can be calculated using:

(7) $$k_{i}=\omega \sqrt{\mu_{i}\varepsilon_{i}}\;$$

$F_{m0}\left(\rho ,z-d\right)$ and $G_{m0}\left(\rho ,z-d\right)$ (*m* = ρ, φ, z) are the direct waves of the electric dipole, $F_{m0}\left(\rho ,z+d\right)$ and $G_{m0}\left(\rho ,z+d\right)$ (*m* = ρ, φ, z) are the ideal reflected waves. $F_{m1}\left(\rho ,z+d\right)$ and $G_{m1}\left(\rho ,z+d\right)$ (*m* = ρ, φ, z) are electric-type waves. $F_{m2}\left(\rho ,z+d\right)$ and $G_{m2}\left(\rho ,z+d\right)$ (*m* = ρ, φ, z) are magnetic-type waves.

The inductance of the wire consists of the internal inductance and the external inductance. The external inductance is calculated using the external magnetic flux. Only the magnetic flux density along the direction that is perpendicular to the plane of the substrate (along the *z* axis in Figure 2) expressed as $B_{0z}$, contributes to the magnetic flux. In the cylindrical coordinates, $B_{0z}$ due to a horizontal electric dipole at (0, 0, d) can be expressed as [18]:

(8) $$\begin{array}{ll} B_{0z}\left(\rho ,\varphi ,z\right)= & \frac{i\mu_{0}Idl}{4\pi }\sin\varphi \{-\left(\frac{\rho }{r_{1}}\right)\left(\frac{k_{0}}{r_{1}}+\frac{i}{r_{1}{}^{2}}\right)e^{ik_{0}r_{1}}+\left(\frac{\rho }{r_{2}}\right)\left(\frac{k_{0}}{r_{2}}+\frac{i}{r_{2}{}^{2}}\right)e^{ik_{0}r_{2}}\\ & +2\pi \underset{j}{\sum }\frac{\lambda_{jB}{{}^{*}}^{2}\tan\gamma_{1B}{}^{*}l}{p^{\prime }\left(\lambda_{jB}{}^{*}\right)}\cdot e^{i\gamma_{0B}{}^{*}\left(z+d\right)}\cdot H_{1}{}^{\left(1\right)}\left(\lambda_{jB}{}^{*}\rho \right)-2k_{0}{}^{2}\sqrt{\frac{1}{\pi k_{0}\rho }}\cdot e^{ik_{0}r_{2}}\\ & \cdot [\sqrt{\frac{\pi }{k_{0}\rho }}-\frac{\pi }{\sqrt{2}}e^{i\frac{\pi }{4}}\cdot T\cdot \exp\left(-i\frac{k_{0}\rho }{2}{\left(\frac{z+d}{\rho }+iT\right)}^{2}\right)\\ & \cdot \mathrm{erfc}\left(\sqrt{-i\frac{k_{0}\rho }{2}{\left(\frac{z+d}{\rho }+iT\right)}^{2}}\right)]\} \end{array}$$

The four terms in the brace of Equation (8) indicate the direct wave, the ideal reflected wave, the magnetic-type trapped surface wave, and the magnetic-type lateral wave of the electric dipole, respectively. The magnetic-type trapped surface wave does not exist when $\sqrt{k_{1}{}^{2}-k_{0}{}^{2}}l<\frac{\pi }{2}$. In this study, the permittivity and the permeability of air are $\varepsilon_{0}=8.85\times 10^{-12}$ F/m and $\mu_{0}=4\pi \times 10^{-7}$ H/m, the permittivity and the permeability of silicon are $\varepsilon_{1}=11.9\times 8.85\times 10^{-12}$ F/m and $\mu_{1}=\mu_{0}=4\pi \times 10^{-7}$ H/m. As the thickness of the substrate is in the order of 100 μm, even when the frequency is as high as 10 GHz, it can be calculated that $\sqrt{k_{1}{}^{2}-k_{0}{}^{2}}l$ is only in the order of 0.1. Thus, the magnetic-type trapped surface wave can be neglected in this study.

In Equation (8), $r_{1}$ is the distance between the source electric dipole at (0, 0, d) and the field point, and $r_{2}$ is the distance between the field point and the ideal image dipole at (0, 0, −d). T can be expressed as:

(9) $$T=\frac{\sqrt{k_{1}{}^{2}-k_{0}{}^{2}}}{k_{0}\tan\sqrt{k_{1}{}^{2}-k_{0}{}^{2}}l}\;$$

The error function “erfc” is defined by [20]:

(10) $$\mathrm{erfc}\left(x\right)=-\int_{x}^{\infty }e^{-t^{2}}dt\;$$

and

(11) $$\mathrm{erfc}\left(\sqrt{x}\right)\approx \frac{1}{\sqrt{\pi x}}e^{-x}\left(1-\frac{1}{2x}+\frac{3}{4x^{2}}+\ldots \right)\;$$

Equation (8) can be rewritten into the form in the rectangular coordinate system as:

(12) $$\begin{array}{ll} B_{0z}\left(x,y,z\right)= & \frac{i\mu_{0}Idl}{4\pi }\cdot \frac{y}{\rho }\\ & \cdot \{-\left(\frac{\rho }{r_{1}}\right)\left(\frac{k_{0}}{r_{1}}+\frac{i}{r_{1}{}^{2}}\right)e^{ik_{0}r_{1}}+\left(\frac{\rho }{r_{2}}\right)\left(\frac{k_{0}}{r_{2}}+\frac{i}{r_{2}{}^{2}}\right)e^{ik_{0}r_{2}}-2k_{0}{}^{2}\sqrt{\frac{1}{\pi k_{0}\rho }}\cdot e^{ik_{0}r_{2}}\\ & \cdot [\sqrt{\frac{\pi }{k_{0}\rho }}-\frac{\pi }{\sqrt{2}}e^{i\frac{\pi }{4}}\cdot T\cdot \exp\left(-i\frac{k_{0}\rho }{2}{\left(\frac{z+d}{\rho }+iT\right)}^{2}\right)\\ & \cdot \mathrm{erfc}\left(\sqrt{-i\frac{k_{0}\rho }{2}{\left(\frac{z+d}{\rho }+iT\right)}^{2}}\right)]\} \end{array}$$

where

(13) $$\rho =\sqrt{x^{2}+y^{2}}\;$$

(14) $$r_{1}=\sqrt{x^{2}+y^{2}+{\left(z-d\right)}^{2}}\;$$

and

(15) $$r_{2}=\sqrt{x^{2}+y^{2}+{\left(z+d\right)}^{2}}\;$$

**Figure 3:**
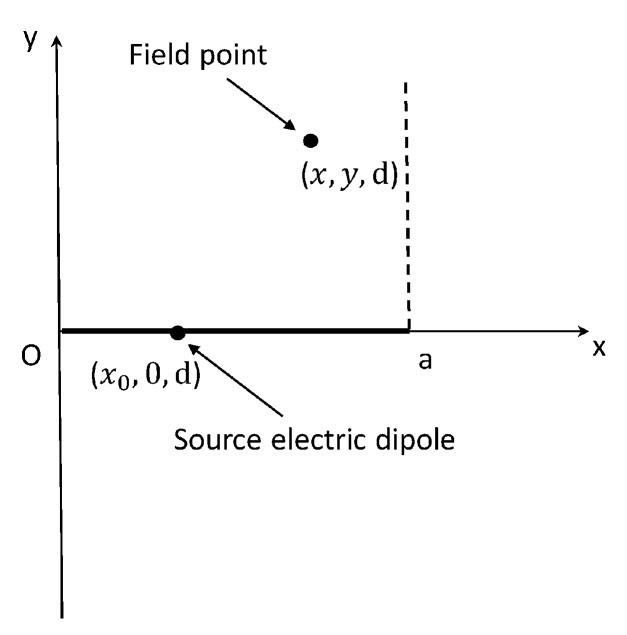
Vertical view of the geometry under consideration.

Figure 3 shows the vertical view of the geometry under consideration.

According to the Equation (12), on (x, y, d) plane, the magnetic flux density $B_{0z}$ due to the wire of length a can be expressed as:

(16) $$B_{0z}\left(x,\;y,d\right)=\int_{0}^{a}\frac{i\mu_{0}I}{4\pi }\cdot \frac{y}{\rho }\cdot \{-\left(\frac{\rho }{r_{1}}\right)\left(\frac{k_{0}}{r_{1}}+\frac{i}{r_{1}{}^{2}}\right)e^{ik_{0}r_{1}}+\left(\frac{\rho }{r_{2}}\right)\left(\frac{k_{0}}{r_{2}}+\frac{i}{r_{2}{}^{2}}\right)e^{ik_{0}r_{2}}-\;2k_{0}{}^{2}\sqrt{\frac{1}{\pi k_{0}\rho }}\cdot e^{ik_{0}r_{2}}\cdot \left[\sqrt{\frac{\pi }{k_{0}\rho }}-\frac{\pi }{\sqrt{2}}e^{i\frac{\pi }{4}}\cdot T\cdot \exp\left(-i\frac{k_{0}\rho }{2}{\left(\frac{2d}{\rho }+iT\right)}^{2}\right)\cdot \;\mathrm{erfc}\left(\sqrt{-i\frac{k_{0}\rho }{2}{\left(\frac{2d}{\rho }+iT\right)}^{2}}\right)]\right\}dx_{0}$$

where

(17) $$\rho =\sqrt{{\left(x-x_{0}\right)}^{2}+y^{2}}\;$$

(18) $$r_{1}=\sqrt{{\left(x-x_{0}\right)}^{2}+y^{2}}\;$$

and

(19) $$r_{2}=\sqrt{{\left(x-x_{0}\right)}^{2}+y^{2}+4d^{2}}\;$$

The external magnetic flux due to the wire can be expressed as:

(20) $$\Psi =\int_{\frac{w}{2}}^{\infty }\int_{0}^{a}B_{0z}dxdy$$

The external inductance of the suspended inductor consisting of a single wire can be calculated using:

(21) $$\;L_{e}=\frac{\Psi }{I}$$

The internal inductance can be calculated using Equation (22) [21]:

(22) $$L_{i}=\frac{a}{\sqrt{wt}}\sqrt{\frac{\mu_{0}}{2\omega \gamma }}\vartheta \;$$

where a, w, t are the length, width, and thickness of the wire. γ is the conductivity of the material of the wire. In this study, the material of the wire was copper. ϑ is a coefficient that is related to w/t and ϑ can be obtained by using a look-up table.

Then the inductance value of the suspended inductor consisting of a single wire can be calculated using (23):

(23) $$\;L=L_{e}+L_{i}\;$$

### 2.2. Calculation of the Inductance Value of the Suspended Inductor Consisting of a Single Rectangular Coil

**Figure 4:**
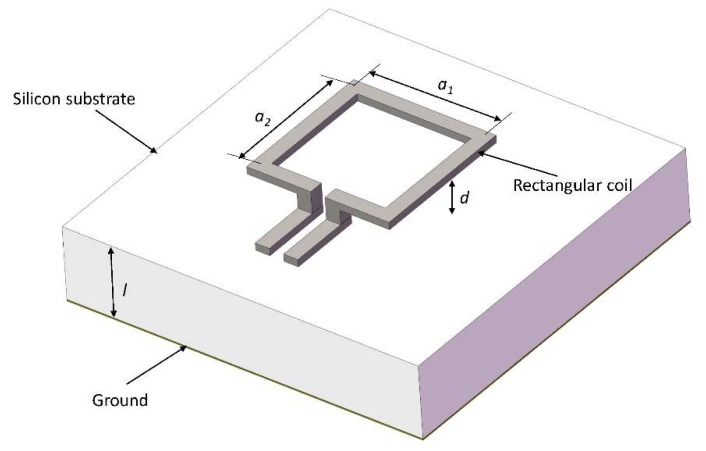
The schematic of the MEMS suspended inductor consisting of a single rectangular coil.

The schematic of the MEMS suspended inductor consisting of a single rectangular coil is shown in Figure 4. The suspension height of the coil is d. The length and width of the rectangular coil are $a_{1}$ and $a_{2}$, respectively. The rectangular coil consists of four wire segments and the magnetic flux density on plane (x, y, d), due to each wire segment, can also be calculated using Equation (16).

Thus, the magnetic flux in the area enclosed by the rectangular coil can be expressed as:

(24) $$\Psi_{coil}=2\times \left(\int_{\frac{w}{2}}^{a_{2}-\frac{w}{2}}\int_{\frac{w}{2}}^{a_{1}-\frac{w}{2}}B_{0z1}dxdy+\int_{\frac{w}{2}}^{a_{1}-\frac{w}{2}}\int_{\frac{w}{2}}^{a_{2}-\frac{w}{2}}B_{0z2}dxdy\right)$$

where $B_{0z1}$ and $B_{0z2}$ are the magnetic flux density due to the wire segment and the length is $a_{1}$ and $a_{2}$, respectively.

Then the external inductance of the suspended inductor consisting of a single rectangular coil can be calculated using:

(25) $$L_{e}=\frac{\Psi_{coil}}{I}$$

The internal inductance of each wire segment can also be calculated using (22). The internal inductance of the rectangular coil can be expressed as:

(26) $$L_{i}=2\times \left(\frac{a_{1}}{\sqrt{wt}}\sqrt{\frac{\mu_{0}}{2\omega \gamma }}\vartheta +\frac{a_{2}}{\sqrt{wt}}\sqrt{\frac{\mu_{0}}{2\omega \gamma }}\vartheta \right)$$

The sum of the external inductance and the internal inductance is the inductance value of the suspended inductor consisting of a single rectangular coil.

## 3. Results and Discussion

In this section, the results calculated using the proposed method are verified using the ANSYS High Frequency Structure Simulator (HFSS) software. Copper is usually used as the structure material of the inductor coil as it has high conductivity. The properties of the copper we used in the simulations were permittivity $\varepsilon_{\mathrm{Cu}}=8.85\times 10^{-12}$ F/m, permeability $\mu_{\mathrm{Cu}}=4\pi \times 10^{-7}$ H/m, and bulk conductivity $\sigma_{\mathrm{Cu}}=5.8\times 10^{7}$ S/m. Considering the fabrication and the influence of the skin effect, the thickness of the existing suspended inductor wire used in GHz is usually around 10 μm [1,2,3,11,22]. The thickness of the wire in this study was 10 μm. The width of the wire in this study was 20 μm. The thickness of the silicon substrate was 300 μm. The ground was considered as the perfect electric conductor.

**Figure 5:**
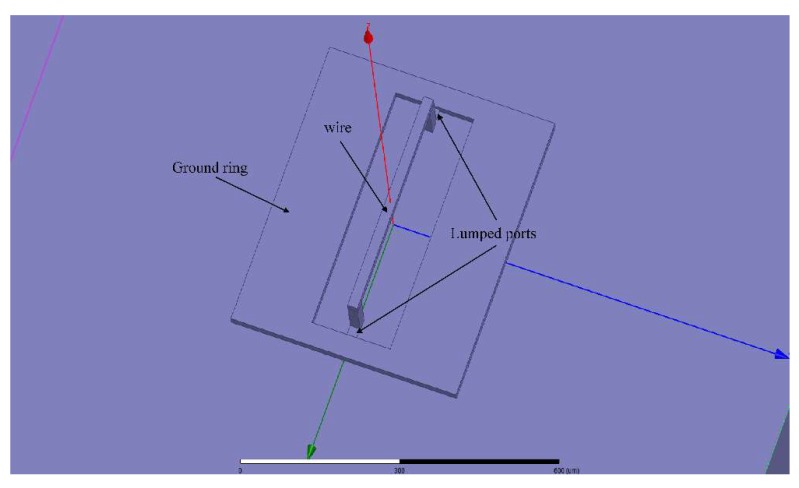
The High Frequency Structure Simulator (HFSS) model of the wire.

First the length of the wire was considered. The inductance values of a series of wires with different lengths were calculated using the method presented in this paper. The lengths of the wires varied from 100 μm to 500 μm. The suspension height of the wire was 50 μm and the frequency was 0.5 GHz. The method in this paper was programmed in MATLAB (The MathWorks, Inc., Natick, MA, USA). The inductance values were also calculated using ANSYS HFSS software. The HFSS model is shown in Figure 5, and lumped ports were chosen. The solution setup was as follows: the maximum number of adaptive solutions was 20 and the maximum delta S was 0.01.

**Figure 6:**
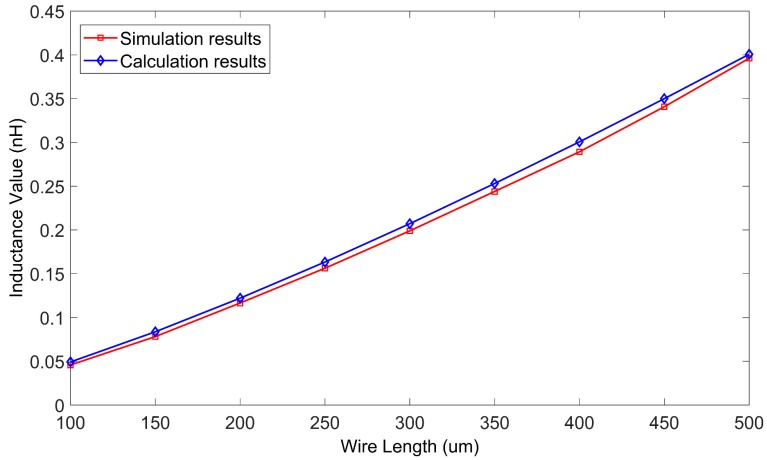
The inductance values as calculated by the proposed method and HFSS, versus wire length.

The computation times of the two methods are listed in Table 1 and the results are shown in Figure 6.

The findings in Table 1 demonstrate that the computation speed of the method in this paper was faster than that of the HFSS simulation. The results in Figure 6 demonstrate that the results calculated by the proposed method supported the HFSS simulation results. The results achieved by HFSS simulation were considered as true values. The maximum and minimum relative deviations of the inductance values were 7.5% and 1.16% when the wire lengths were 100 μm and 500 μm, respectively.

**Figure 7:**
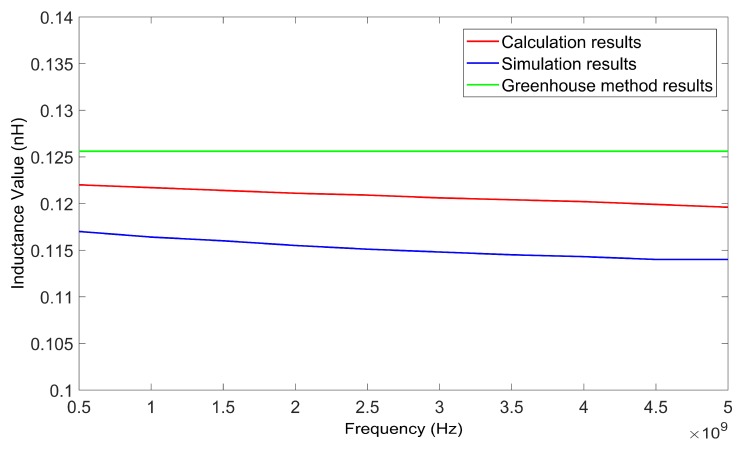
Inductance values of 200 μm wire as calculated by the proposed method, HFSS, and the Greenhouse method, versus frequency.

**Figure 8:**
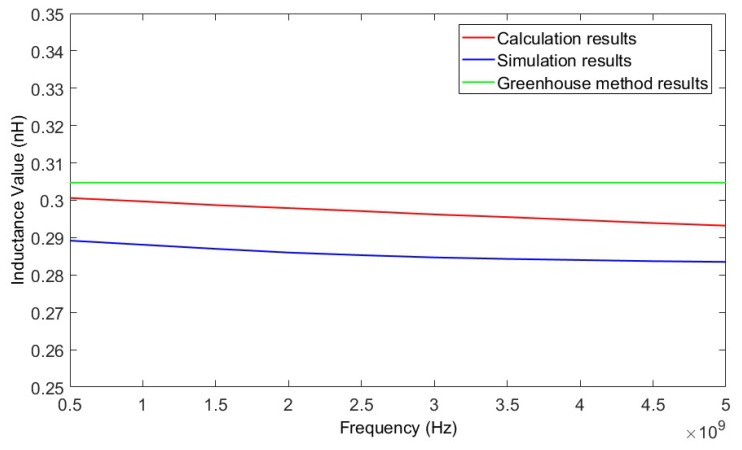
Inductance values of 400 μm wire as calculated by the proposed method, HFSS, and the Greenhouse method, versus frequency.

The inductance values of the inductor wire at different frequencies can be calculated using the method in this paper. The suspension height of the wire was 50 μm. By changing the frequency from 0.5 GHz to 5 GHz, a series of inductance values for two kinds of wires, with lengths of 200 μm and 400 μm, was obtained. Figure 7 and Figure 8 show the calculated results of the two wires as verified by HFSS. The results calculated using the Greenhouse method are also shown in Figure 7 and Figure 8 for comparison.

From Figure 7 and Figure 8 it can be determined that the results achieved using the method in this paper agreed with the simulation results better than the results calculated using the Greenhouse method. As the results achieved using the HFSS simulation were considered as true values, the relative deviation of the inductance values calculated using the method in this paper were all less than 5.2%.

**Figure 9:**
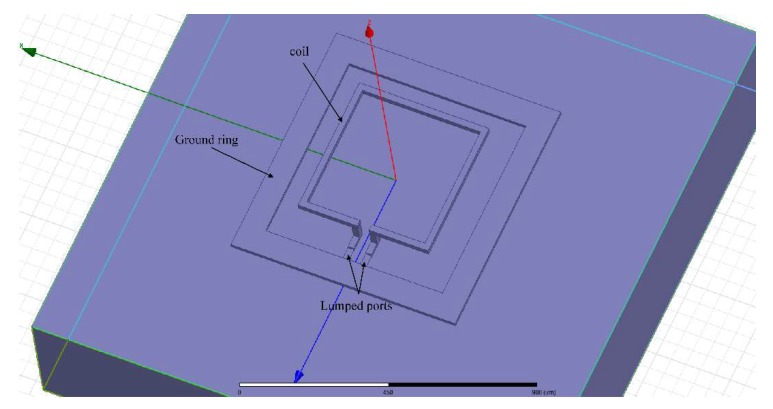
The HFSS model of the single turn suspended inductor.

Therefore, two kinds of single turn inductors were chosen to verify the accuracy of the theoretical calculation results. The side lengths of the two square coils were 400 μm and 500 μm. The suspension height of both inductors was 50 μm. The frequency varied from 0.5 GHz to 6 GHz and the inductance value was calculated every 500 MHz using the method in this paper in the MATLAB software. The HFSS model of the single turn suspended inductor is shown in Figure 9, and lumped ports were chosen. The solution setup was as follows: the maximum number of adaptive solutions was 20 and the maximum delta S was 0.01.

**Figure 10:**
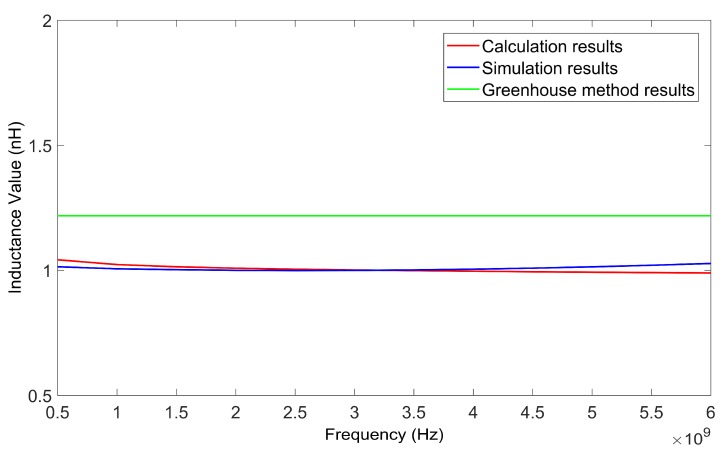
Inductance values of a single turn coil inductor (side length of 400 μm) as calculated by the proposed method, HFSS, and the Greenhouse method, versus frequency.

**Figure 11:**
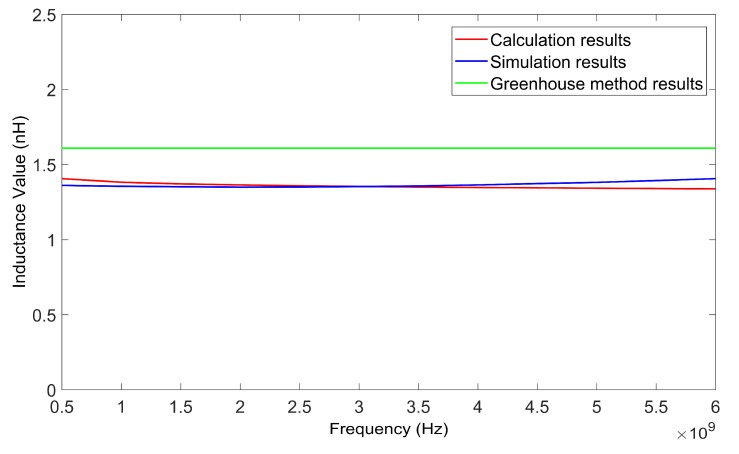
Inductance values of a single turn coil inductor (side length of 500 μm) as calculated by the proposed method, HFSS, and the Greenhouse method, versus frequency.

Table 2 presents the computation times of the two methods. Figure 10 and Figure 11 show the calculated results of the two inductors, as verified by HFSS respectively. The inductance values of the two inductors calculated with the Greenhouse method are also shown in Figure 10 and Figure 11 for comparison.

Table 2 also shows that the computation speed of the method in this paper was faster than that of the HFSS simulation. Figure 10 and Figure 11 show that the calculation results agree with the simulation results, better than the results calculated using the Greenhouse method. When the frequency varied from 0.5 GHz to 6 GHz, the relative deviations of the calculation results using the method of this paper were less than 5% for both single turn coil inductors. As the frequency increased, the relative deviation became higher due to the skin effect.

The skin effect is a complex issue that has an influence on the performance of the inductors. The skin effect results in a current flowing in the outer area of the conductor when the frequency is high. As for inductors with a single square coil, the current density is greater at the inner corners of the square coil when the frequency is high. The skin depth *δ* is the effective depth of the penetration of the current, which can be calculated using:

(27) $$\delta =\frac{1}{\sqrt{\pi f\mu \sigma }}$$

where *f*, *μ* and *σ* represent the frequency, permeability, and conductivity. The skin depth of copper at different frequencies can be calculated using (27), the results of which are shown in Table 3.

From Table 3 we can find that the skin effect is more significant when the frequency is higher. When the frequency is lower than 3 GHz, the influence of the skin effect on the 10 μm thickness wire is not highly significant. When the frequency is higher than 5 GHz, the skin depth is less than 1 μm. Thus, the relative deviations become higher as the frequency increases.

**Figure 12:**
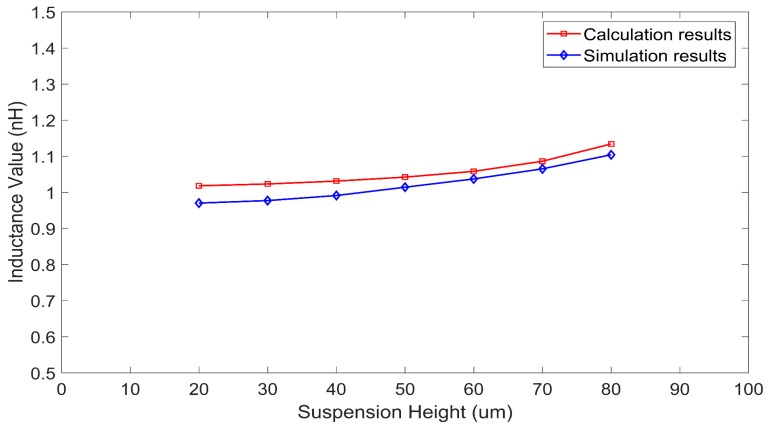
Inductance values of a single turn coil inductor (side length of 400 μm) as calculated by the proposed method and HFSS, versus suspension height.

**Figure 13:**
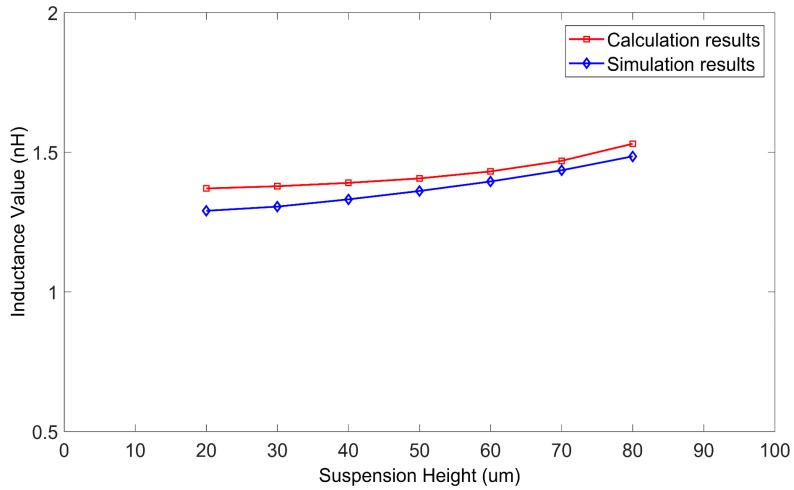
Inductance values of a single turn coil inductor (side length of 500 μm) as calculated by the proposed method and HFSS, versus suspension height.

Figure 12 and Figure 13 show the curves of the inductance values of the two single turn coil inductors varying with the suspension height. The suspension height varied from 20 μm to 80 μm. The side length of the two inductors were 400 μm and 500 μm, and the frequency was 0.5 GHz.

As Figure 12 and Figure 13 show, the inductance values obtained by the proposed method agreed well with those obtained by HFSS simulation. The relative deviations were all less than 6.5%.

The substrate loss caused by the silicon substrate led to a reduction in the inductance value of the inductors. Thus the actual inductance value of an inductor with a silicon substrate was lower than the theoretical inductance value of an inductor coil calculated using the Greenhouse method (see Figure 7, Figure 8, Figure 10, and Figure 11). Substrate loss was caused by the conductive nature of the silicon substrate, which can be effectively reduced by lifting the suspension height of the coil [12]. As the suspension height increased, the inductance value also increased (see Figure 12 and Figure 13). Thus, compared to the Greenhouse method, the method in this paper took into account the influence of the substrate and the suspension height. Compared to HFSS simulation, the computation speed of this method was faster. Furthermore, it can be used to calculate the variation in inductance values during a process of suspended inductor deformation, such as under mechanical shock.

## 4. Conclusions

In this paper, a method was proposed to calculate the inductance values of microelectromechanical system (MEMS) suspended inductor wires, which are the basic component of the MEMS suspended inductor coil with a silicon substrate. The influence of the silicon substrate and the suspension height were considered by this method. The inductor wires were considered as a series of electric dipoles in stratified media, and the inductance values were obtained by solving the electromagnetic fields generated by the dipoles. The calculation process was presented. Following this, the method was extended to a MEMS suspended single turn inductor with a silicon substrate. Several single wire suspended inductors and two kinds of single turn coil inductors, were used as examples and the ANSYS High Frequency Structure Simulator (HFSS) software was used to verify the accuracy of the results obtained using the proposed method. The results achieved using the method in this paper were also compared to those calculated by the Greenhouse method, to demonstrate the influence of the silicon substrate and the suspension height of the MEMS suspended inductor. The calculation results obtained using the method in this paper were found to agree well with the simulation results, and the computation speed of this method was faster than the HFSS simulation. We found that that the substrate loss caused by the silicon substrate led to a reduction of the inductance value of the inductors. By lifting the suspension height of the coil, the MEMS suspended inductor achieved a higher inductance value because the substrate loss was reduced. Therefore, using the method in this paper we calculated the variation of the inductance value directly, without simulation or measurement, when the deformation variation of the MEMS suspended inductors during shock was obtained.

## Figures and Tables

**Table 1:** Time to calculate the inductance values of wires using each method.

Length of the Wire/μm	Computation Time of the Method in this Paper/s	Computation Time of HFSS/s
100	10.82	43.81
150	11.72	46.39
200	19.55	46.67
250	22.58	46.14
300	24.98	46.66
350	26.05	45.82
400	28.66	49.68
450	30.09	50.10
500	31.45	56.04

**Table 2:** Time to calculate the inductance values of single turn suspended inductors using each method.

Side Length of the Wire/μm	Computation Time of the Method in this Paper/s	Computation Time of HFSS/s
400	44.26	72.24
500	44.95	80.86

**Table 3:** Skin depth of copper.

Frequency/GHz	Skin Depth/μm
0.1	6.61
0.5	2.96
1	2.09
3	1.21
5	0.94
7	0.79
9	0.69
10	0.66
